# Bleb Revision Using an L-shaped Conjunctival Flap Posterior to the Limbus in Eyes With Bleb Failure After PreserFlo MicroShunt Implantation

**DOI:** 10.7759/cureus.106575

**Published:** 2026-04-07

**Authors:** Katsuhiko Maruyama

**Affiliations:** 1 Ophthalmology, Yashio Maruyama Eye Clinic, Saitama, JPN

**Keywords:** bleb failure, bleb revision, flow redirection, glaucoma surgery, posterior conjunctival flap, preserflo® microshunt, preserflo microshunt complications

## Abstract

Bleb fibrosis is a major cause of early surgical failure after PreserFlo MicroShunt implantation. Although needling is commonly attempted as first-line management, its effectiveness may be limited in cases with dense Tenon’s encapsulation. The optimal surgical approach for open revision has not been well defined. This case series presents four consecutive eyes with bleb failure after PreserFlo MicroShunt implantation treated with revision using a fornix-based L-shaped conjunctival flap posterior to the limbus technique. Intraocular pressure (IOP), best-corrected visual acuity, corneal endothelial cell density, complications, and need for additional interventions were evaluated. The mean IOP decreased from 30.4 mmHg preoperatively to 9.5 mmHg at one month, 10.8 mmHg at three months, 12.5 mmHg at six months, and 13.4 mmHg at one year postoperatively. Visual acuity remained stable, and no clinically significant endothelial cell loss was observed. One transient choroidal detachment resolved spontaneously. One case required adjunctive topical glaucoma medication during follow-up; however, no patient required additional surgical intervention. Bleb revision using this posterior conjunctival flap technique effectively restored filtration and achieved sustained IOP reduction in eyes with bleb failure after PreserFlo MicroShunt implantation. By avoiding conjunctival dissection directly over the implant and minimizing limbal manipulation, this flow-redirection technique may preserve conjunctival integrity and represent a rational surgical alternative in selected cases.

## Introduction

The PreserFlo MicroShunt (PMS) (Santen Pharmaceutical, Co., Ltd., Osaka, Japan) is a glaucoma surgical device measuring 8.5 mm in length with a lumen diameter of 70 μm. It is made from a highly biocompatible and bioinert material called poly(styrene-block-isobutylene-block-styrene) (SIBS). PMS is used in filtration surgery to reduce intraocular pressure (IOP) by draining aqueous humor from the anterior chamber into the subconjunctival space [1]. Although PMS implantation is less effective than trabeculectomy at lowering IOP [2,3], it may benefit patients due to a lower risk of hypotony-related events [2] or a lower reintervention rate [3]. As PMS implantation does not require the creation of a scleral flap, an ostomy, or a peripheral iridectomy and the subsequent suturing of the scleral flap, as is necessary for trabeculectomy, it is also a less invasive and more standardized procedure [4]. Consequently, PMS implantation is increasingly being performed as an alternative to trabeculectomy in Japan [5].

As PMS implantation involves a filtration surgery, a certain percentage of eyes are destined to develop filtration failure due to fibrosis of scar tissue after surgery. There are several methods that can be used to manage increased IOP postoperatively, including steroids, non-steroidal anti-inflammatory drugs, IOP-lowering medications, bleb needling, and open revision [6,7]. Of these methods, needling is commonly performed as a primary intervention to restore filtration. However, its efficacy may be limited, particularly when dense Tenon’s fibrosis surrounds the distal end of the tube [8]. This fibrosis is primarily attributed to activation of fibroblasts within Tenon’s capsule, leading to extracellular matrix deposition and encapsulation of the filtering bleb. In such cases, open bleb revision may be considered a secondary intervention when needling is insufficient. Although several reports have described open revision following filtration failure after PMS implantation [9,10], detailed discussion regarding the optimal conjunctival incision site and surgical concept remains limited.

In previously reported techniques, conjunctival incision is often performed directly over the implant [10]. However, direct dissection above the device may promote additional scarring in the region most critical for long-term filtration and may compromise future surgical options. I, therefore, developed a fornix-based L-shaped conjunctival flap posterior to the limbus technique designed to release fibrotic obstruction around the distal extremity of the tube and redirect aqueous humor posteriorly without dissecting directly over the implant. This study aimed to evaluate the short-term efficacy and safety of this posterior L-shaped conjunctival flap revision technique in eyes with bleb failure after PMS implantation.

## Case presentation

Subjects

This retrospective case series included four consecutive eyes with bleb failure after PMS implantation. All revision procedures were performed by the same surgeon at Yashio Maruyama Eye Clinic between July 2023 and January 2024. All patients were followed for at least 12 months. This study was approved by the Ethics Review Committee of the Japan Medical Association (No. R5-14). Table 1 shows the patient characteristics at baseline.

**Table 1:** Patient characteristics at baseline. *: The time between PreserFlo MicroShunt implantation and bleb revision. **: Mean of three measurements. M: male; F: female; PEG: pseudoexfoliation glaucoma; POAG: primary open-angle glaucoma; PMS: PreserFlo MicroShunt implantation; BR: bleb revision; IOP: intraocular pressure; BCVA: best-corrected visual acuity; ECD: endothelial cell density

Case number	Age	Sex	Diagnosis	PMS-BR duration^*^	IOP^**^	BCVA	ECD
				(week)	(mmHg)	(logMAR)	(cells/mm²)
1	79	M	PEG	6	41.3	0.3	2,103
2	81	F	POAG	12	18.3	0.1	2,563
3	55	M	POAG	6	35.0	0.1	2,567
4	84	F	PEG	11	27.0	0.4	2,261

The time between PMS implantation and bleb revision was 8.8 weeks (range = 6 to 12 weeks). Before bleb revision, the mean IOP of three measurements was 30.4 mmHg (18.3 to 41.3 mmHg), the mean best-corrected visual acuity (logMAR) was 0.2 (0.1 to 0.4), and the mean corneal endothelial cell density was 2,373.5 cells/mm² (2,103 to 2,567 cells/mm²). All eyes were pseudophakic and had undergone PMS implantation within the preceding three months. All cases had received needling before revision surgery. In all cases, the filtering blebs showed encapsulation without marked neovascularization, consistent with bleb failure due to fibrotic changes. No glaucoma medications were used before revision.

Method

**Figure 1:**
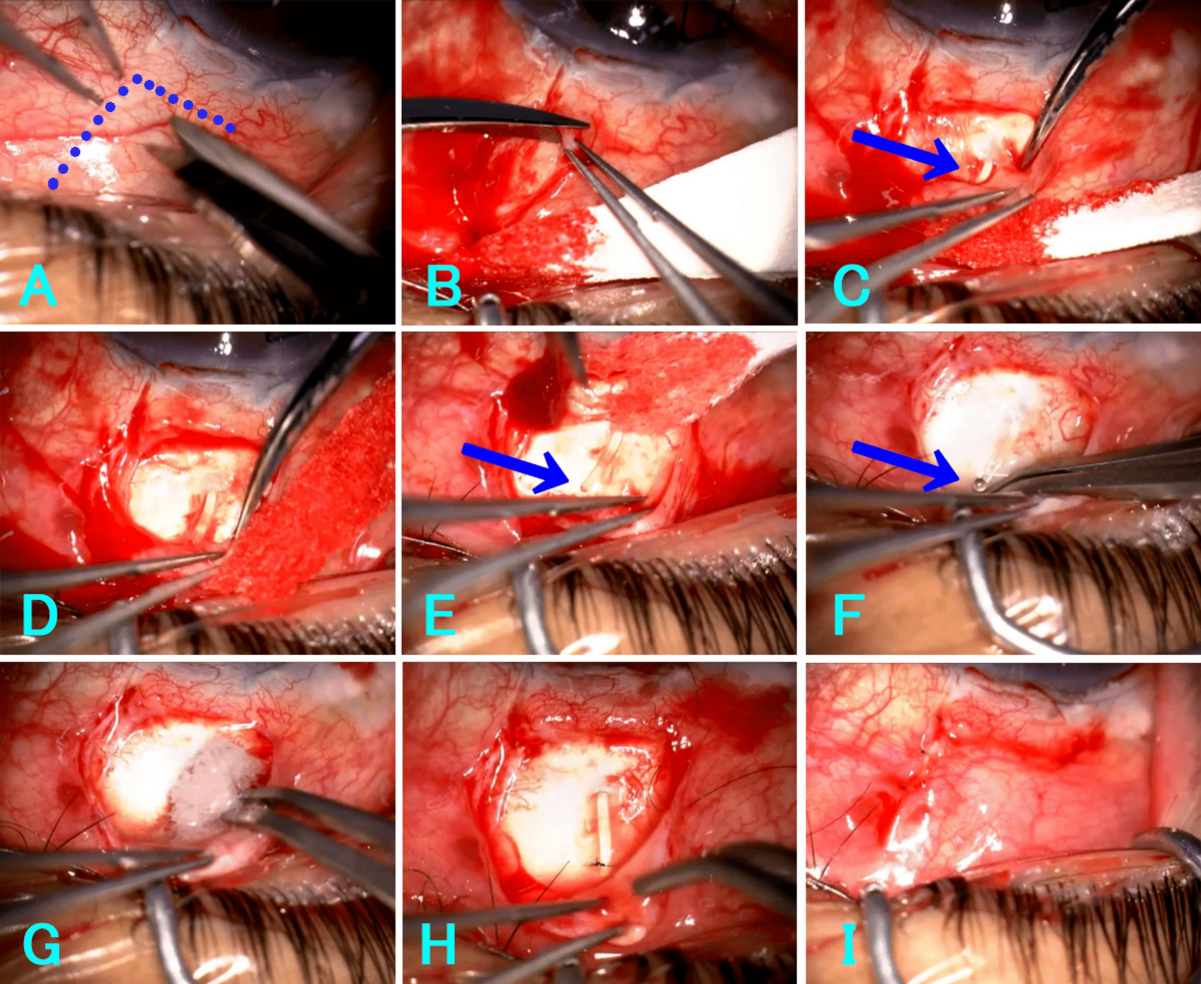
Posterior L-shaped conjunctival flap revision technique. Intraoperative findings in Case 3. (A) Pre-incision view. The dotted line indicates the planned incision line. (B) Creation of a posterior L-shaped conjunctival flap 2 mm posterior to the limbus. (C) Identification of the PreserFlo MicroShunt (arrow) after dissection of Tenon’s capsule. (D) Posterior dissection of Tenon’s capsule toward the fornix. (E) Release of fibrotic tissue surrounding the distal extremity of the tube (arrow). (F) Confirmation of aqueous humor outflow from the distal tip (arrow). (G) Application of 0.04% mitomycin C under the conjunctival flap. (H) Fixation of the posterior edge of the tube to the sclera with 10-0 nylon. (I) Closure of conjunctiva and Tenon’s capsule.

The same surgical technique was applied in all cases (Figure 1, Video 1). After topical anesthesia with 4% lidocaine eye drops (Xylocaine 4%, Sandoz Pharma K.K., Tokyo, Japan), 1.5 mL of 2% lidocaine (Xylocaine for Intravenous Injection 2%, Sandoz Pharma K.K., Tokyo, Japan) was injected into the sub-Tenon’s space. A posterior fornix-based L-shaped conjunctival flap was created radially and parallel to the limbus at approximately 2 mm posterior to the limbus. Tenon’s capsule and connective tissue were dissected en bloc from the sclera using microscissors, proceeding posteriorly toward the fornix. The distal extremity of the tube was identified and carefully released from surrounding fibrotic tissue. Adequate aqueous humor outflow from the distal tip was confirmed intraoperatively. Mitomycin C (Mitomycin 2 mg for Ophthalmic Topical Use, Kyowa Hakko Kirin Co. Ltd., Tokyo, Japan) 0.04% (0.4 mg/mL) was applied beneath the conjunctival flap for three minutes using soaked surgical sponges. After thorough irrigation with balanced salt solution (BSS Plus, Alcon, Tokyo, Japan), the posterior edge of the tube was secured to the sclera using 10-0 nylon (10-0 Nylon Black Mono, with needle; code 1404, Mani Co. Ltd., Tochigi, Japan). The conjunctival flap, including Tenon’s capsule, was then closed with 10-0 nylon sutures.

**Video 1:** Posterior L-shaped conjunctival flap revision technique. Intraoperative findings in Case 3.

Topical 1.5% levofloxacin (Cravit, Santen Pharmaceutical) and 0.1% betamethasone (Rinderon, Shionogi Pharma Co. Ltd., Osaka, Japan) were administered three times daily for three weeks postoperatively.

Results

**Figure 2:**
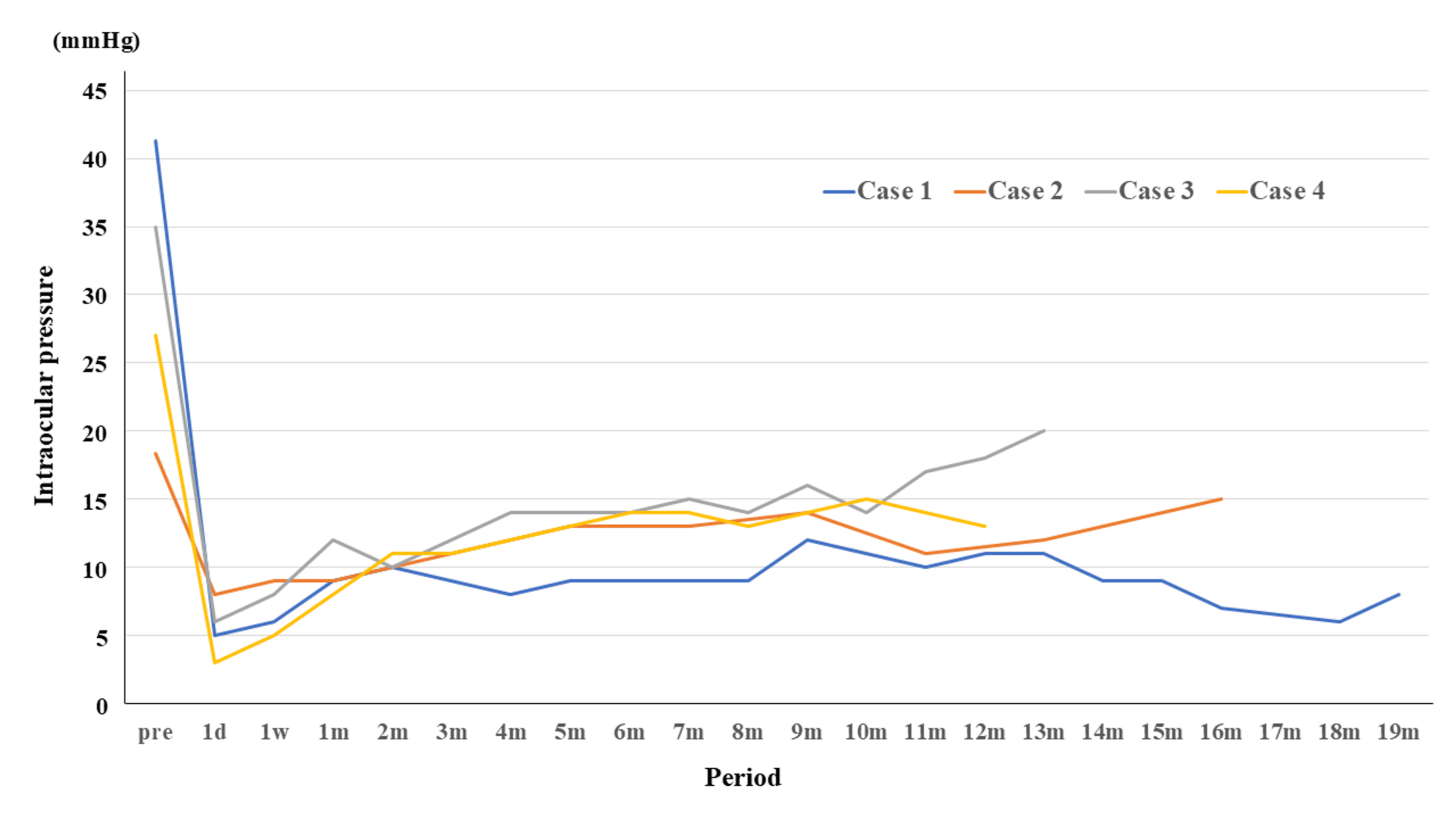
Intraocular pressure changes in four cases before and after revision surgery. d: day; w: week; m: month

No complications such as conjunctival injury or wound dehiscence occurred during the bleb revision. No early postoperative leakage from the conjunctival incision site was observed. The IOP changes in each eye are shown in Figure 2, and the individual clinical course is shown in Table 2.

**Table 2:** Individual clinical course. *: Improved for three weeks without treatment. **: Mean of three measurements. IOP: intraocular pressure; BCVA: best-corrected visual acuity; ECD: endothelial cell density

Case number	Postoperative one year			Follow-up period	Complication
	IOP^**^	BCVA	ECD		
	(mmHg)	(logMAR)	(cells/mm²)	(month)	
1	11	0	2,009	19	Choroidal detachment^*^
2	12	0.2	2,251	16	-
3	18	0	2,699	13	-
4	13	0.4	1,651	12	-

Mean IOP decreased to 9.5 mmHg (range = 8 to 12 mmHg) at one month, 10.8 mmHg (range = 9 to 12 mmHg) at three months, 12.5 mmHg (range = 9 to 14 mmHg) at six months, and 13.4 mmHg (range = 11 to 18 mmHg) at one year postoperatively. Visual acuity remained stable throughout follow-up. No clinically significant endothelial cell loss was observed. One patient developed transient choroidal detachment, which resolved spontaneously within three weeks. The eye of Case 3 required an addition of IOP-lowering medication after nine months postoperatively; however, no patient required additional surgical intervention.

**Figure 3:**
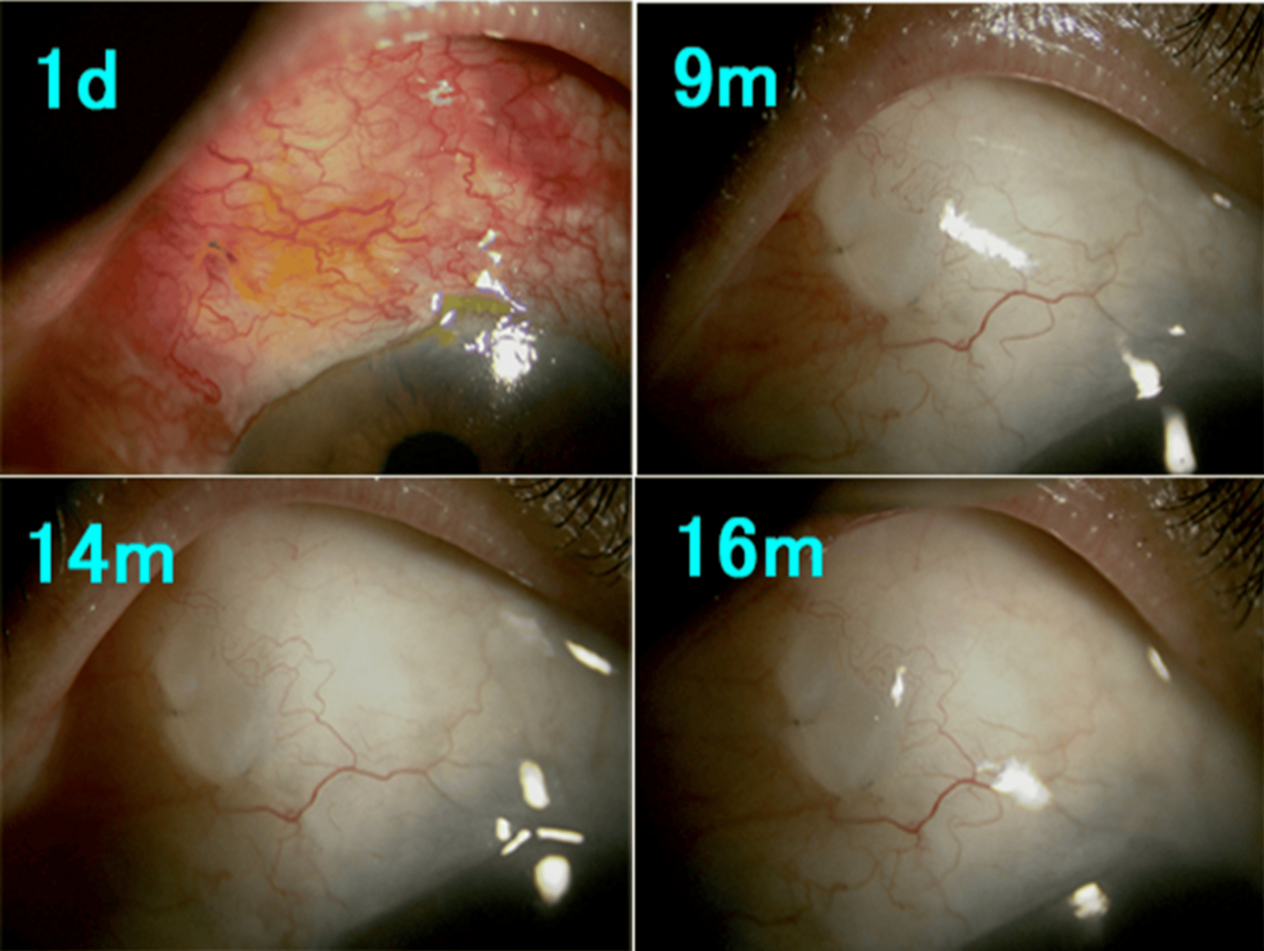
Slit-lamp microscopy findings after bleb revision in Case 1. Diffuse posterior bleb formation toward the fornix with minimal vascularization was observed during follow-up. d: day; m: month

**Figure 4:**
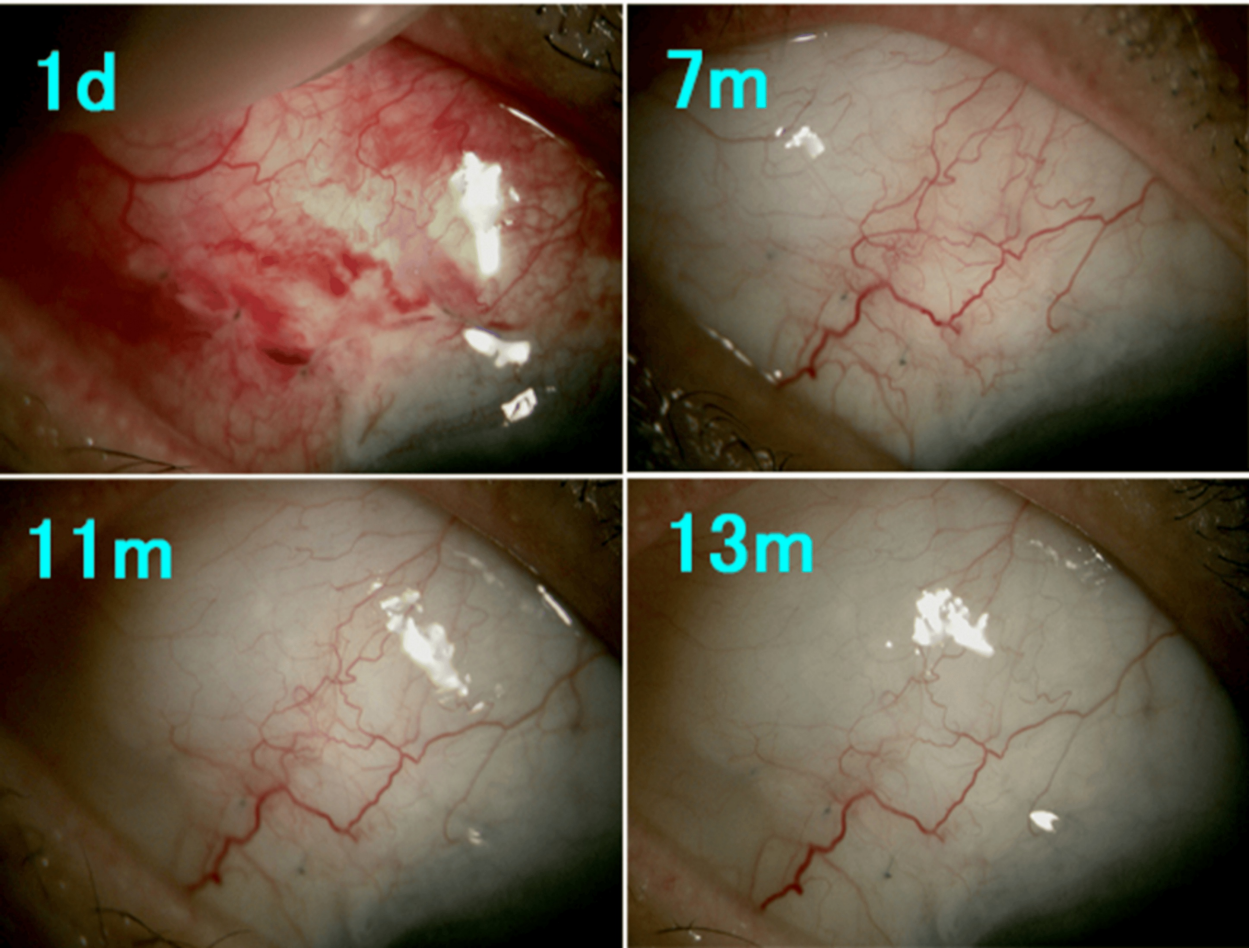
Slit-lamp microscopy findings after bleb revision in Case 2. Diffuse posterior bleb formation toward the fornix with minimal vascularization was observed during follow-up. d: day; m: month

**Figure 5:**
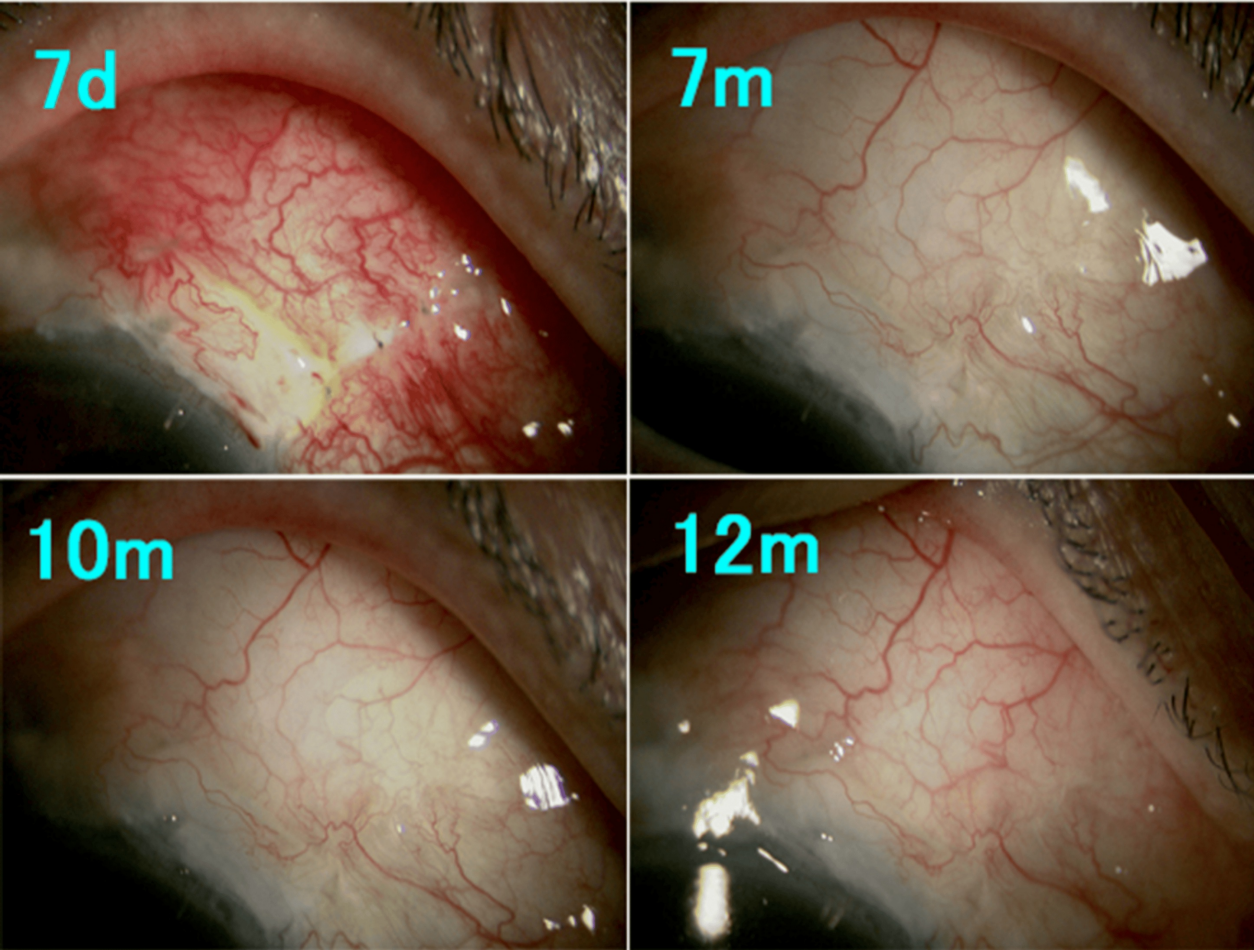
Slit-lamp microscopy findings after bleb revision in Case 3. Posterior bleb formation was observed; however, adjunctive intraocular pressure-lowering medication was initiated at nine months, after which increased vascularization of the bleb was noted. d: day; m: month

**Figure 6:**
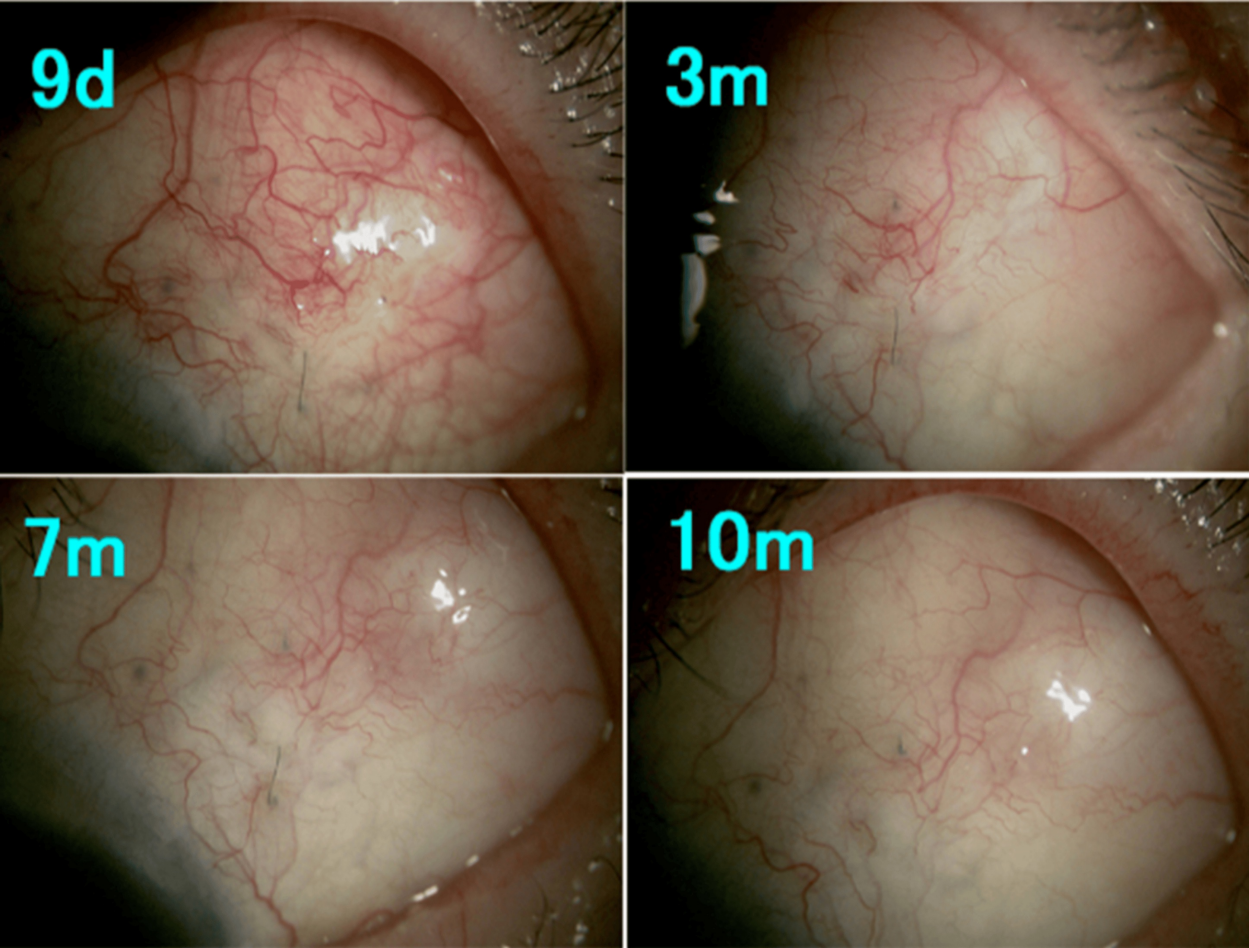
Slit-lamp microscopy findings after bleb revision in Case 4. Diffuse posterior bleb formation toward the fornix with minimal vascularization was observed during follow-up. d: day; m: month

Figures 3-6 show postoperative slit-lamp findings in Cases 1-4. Diffuse posterior bleb formation was observed in most cases during follow-up. No ocular infection occurred throughout the entire clinical course.

## Discussion

This study evaluated the efficacy and safety of a posterior L-shaped conjunctival flap revision procedure for treating bleb failure after PMS implantation, with the conjunctival incision placed posterior to the limbus. All cases achieved a sustained reduction in IOP compared to preoperative levels, and, in most cases, no additional topical medications or surgery were necessary. These results suggest that this technique may be clinically significant.

Bleb fibrosis is a known cause of failure after bleb-forming procedures. In PMS implantation, aqueous humor is directed posteriorly beneath Tenon’s capsule. Therefore, fibrosis around the distal extremity of the device can severely restrict flow. Although needling is less invasive, it may be insufficient when dense fibrosis surrounds the distal tip of the tube. Open bleb revision allows for direct visualization and release of obstructing tissue. European physicians with extensive experience with PMS implantation agree that open revision is preferred over bleb needling when the target IOP is not maintained or when filtration failure occurs postoperatively [6,7]. Strzalkowska et al. reported that open bleb revision demonstrated substantial IOP reduction and a favorable safety profile [10]. The mean IOP decreased from 26.4 mmHg pre-revision to 15.9 mmHg at 12 months post-revision. Most complications were mild and managed conservatively. However, the reported techniques involve a conjunctival incision directly over the implant. Direct dissection at this critical filtration site may induce additional scarring.

The key concept of this technique is posterior flow redirection. Importantly, the novelty of this technique lies not in the incision site alone, but in the combination of posterior flow redirection and preservation of conjunctival tissue over the implant. To our knowledge, the posterior L-shaped conjunctival flap design described in this study has not been previously reported for revision surgery after PMS implantation. This design was developed to minimize conjunctival manipulation over the implant and to facilitate posterior filtration while preserving conjunctival tissue near the limbus. By creating a fornix-based L-shaped conjunctival flap 2 mm posterior to the limbus and dissecting Tenon’s capsule toward the fornix, fibrotic obstruction can be released without exposing sclera adjacent to the limbus or dissecting directly above the implant. This posterior incision site was intentionally selected to avoid manipulation of conjunctival tissue near the limbus, where prior surgical scarring is more likely to be present, to reduce the risk of conjunctival damage, and to preserve tissue for potential future filtration procedures. This approach may preserve conjunctival integrity around the device and reduce the risk of further scarring. Consistent with this concept, postoperative slit-lamp findings showed diffuse posterior bleb formation toward the fornix in most eyes, suggesting that the intended posterior flow redirection was achieved.

Fixation of the posterior edge of the tube to the sclera may further stabilize posterior aqueous flow and reduce the likelihood of re-encapsulation. During the revision process, the distal end of the tube may become displaced or surrounded by fibrotic tissue. Fixation of the posterior edge of the tube to the sclera may help maintain its posterior orientation and stabilize aqueous outflow, thereby reducing the likelihood of re-encapsulation.

Although excision of scar tissue was not routinely performed in this case series, the necessity of this procedure warrants further consideration. In Case 3, the filtering function deteriorated one year after surgery, requiring the use of anti-glaucoma medications. If scar tissue had been excised, filtration function might have been maintained. However, satisfactory IOP was achieved in the other three eyes despite the same surgical technique being performed. Further investigation is needed as the number of cases increases.

An alternative approach would be to reopen the conjunctiva anteriorly toward the limbus, similar to the initial surgery. While this may also restore filtration, there may be specific disadvantages. In eyes with previous cataract surgery or prior filtering procedures, scarring near the limbus may increase the risk of conjunctival damage during dissection. Additionally, in cases with dense limbal scarring, advancing and repositioning the conjunctiva toward the limbus may be technically difficult. By avoiding limbal manipulation, my posterior approach may reduce the risk of conjunctival injury and preserve tissue for potential future surgical interventions. Although the conjunctival incision is created closer to the distal end of the tube, where aqueous humor exits, the direction of aqueous humor outflow is oriented posteriorly away from the incision site. Therefore, the risk of leakage from the incision is considered to be low, which is consistent with our clinical findings showing no early postoperative leakage.

This study has several limitations, including its retrospective design, small sample size, and lack of a control group. Objective bleb morphology assessment using anterior-segment optical coherence tomography or ultrasound biomicroscopy was not performed. Larger comparative studies with longer follow-up are needed to confirm the long-term benefits of this technique.

Nevertheless, to our knowledge, there are limited detailed descriptions of conjunctival incision strategies for revision surgery after PMS implantation. The findings of this report suggest that posterior L-shaped conjunctival flap revision represents a rational and potentially advantageous surgical option in selected cases.

## Conclusions

Posterior L-shaped conjunctival flap revision was associated with restoration of filtration and sustained IOP reduction in eyes with bleb failure after PMS implantation in the short term. By avoiding conjunctival dissection directly over the implant and minimizing limbal manipulation, this flow-redirection technique may help preserve conjunctival integrity and may represent a promising surgical option in selected cases. These findings are preliminary, and further studies with larger cohorts and longer follow-up are warranted.
